# Formulation, Development and Evaluation of Budesonide Oral Nano-sponges Using DOE Approach: *In Vivo* Evidences

**DOI:** 10.34172/apb.2021.041

**Published:** 2020-08-05

**Authors:** Amarjit Salunke, Neeraj Upmanyu

**Affiliations:** School of Pharmacy and Research People’s University Bhopal- 462037, MP, India.

**Keywords:** Budesonide nano-sponges, Colon tissue, Box-Behnken design, Quasi-emulsion solvent, Inflammatory bowel disease, Eudragit S-100

## Abstract

***Purpose:*** The prevalent types of idiopathic inflammatory bowel disease are ulcerative colitis (UC) and Crohn’s disease, which affects a large number of populations. Budesonide (BUD) is a glucocorticoid with potent anti-inflammatory activity but low systemic efficacy because of high receptor affinity and rapid diversion. To overcome low efficacy and availability, a novel BUD nano-sponges was formulated using quasi- solvent diffusion and Eudragit S-100 as polymer. It was then investigated for the effect of process variables using Box-Behnken design.

***Methods:*** The BUD Nano sponges were evaluated for particle size, particle size, polydispersity, percent drug entrapment, drug release pattern. The formulation was evaluated by an *in vivo* study using male Wistar rats and parameters such as clinical activity score, colon/body weight ratio (C/B ratio), macroscopic ulceration (damage score) activity were performed. Finally, histopathological examination was performed on colon tissue samples.

***Results:*** The formulation showed better efficacy and availability as compared with the available formulations of BUD, which indicates the good efficacy of the formulated nanosponges. The clinical activity score was attenuated by the formulated nanosponges in the Wistar rats. The colon to body weight ratio was significantly reduced as compared with the control formulation. The histopathology of colon treated with nanosponges showed normal structure and architecture of the colon.

***Conclusion:*** The results of the present work confirmed the utility of BUD nano-sponges as novel carriers in management IBD.

## Introduction

Ulcerative colitis (UC) and Crohn’s disease are the disorders collectively known as inflammatory bowel disease (IBD). While Crohn’s disease can influence every part of the intestinal part and frequently affecting the colon and distal ileum, while Crohn’s disease influences only the colon.^1^ The two types of IBD are highlighted by exacerbated uncontrolled intestinal inflammation that prompts low quality of life and requires delayed therapeutic or surgical interventions.^2^ In Europe, IBD affecting more than 2.5 million peoples (~0.5%) and is getting progressively common in Asia and emergent nations.^3^ The frequency of IBD is expanding, specifically emergent nations. Even though the etiology of these inflammatory disorders isn’t completely understood, there is a developing body of evidence that morbidity of IBD is related to a hereditary inclination. The additional factors might be related to unsettling influence in the immune system, and irregular intestinal microflora (quantitatively and qualitatively), which has been affirmed in murine models of IBD.^4,5^ The significance of these complex interactions results in disturbed intestinal homeostasis and an unbalanced inflammatory microflora. Crohn’s disease is characterized by a transmural inflammation that may influence the layers of the GI wall, while UC is a mucosal inflammation and delineated to the colon.^6,7^

There is a developing enthusiasm for multi-particulate modified delivery systems, particularly for site-specific targeting of the gastrointestinal tract. The systems of modified release were very complex, and their huge scale assembling requires numerous abilities and innovative advancement.^8,9^ Among the various kinds of different unit dosage forms, nano-sponges shown up very fascinating dose structures from the monetary process advancement and scale-up perspectives. Nano-sponges having a colloidal structure in which a small solid particle is incorporated in the cavities and mesh-like system- to encapsulate wide assortments of molecules like anti-cancer, proteins, DNA, etc.^10,11^ Nano-sponges are 3D systems with a backbone of the naturally long-length polymer. The nano-sponges are prepared by an interaction between cross-linking polyesters and peptides, contrasted with a few other nano dimensions medicate drug delivery systems. Nano-sponges are lipid in nature, and also, they can scatter in the aqueous transporting fluid. They can be used to overcome the bitter taste of drugs. The medication discharge from the nano-sponge system can be changed by modifying cross-linker to polymer proportions. Nano-sponge binds to the surface of the target site in their circulation process in the body and discharges the medication in a controlled and anticipated way.^12-15^ Budesonide (BUD) is the locally acting corticosteroid with a brilliant affinity for glucocorticoid receptors with the strong anti-inflammatory activity. It offers many benefits over old steroids. BUD has 200 times higher topical potency than hydrocortisone and only 10 % of systemic bioavailability. It was reported that BUD showed less systemic side effects than prednisone. Favorably BUD is an ideal drug for the local therapy of IBD include low oral bioavailability, quick clearance and toxic metabolites.^1,16,17^ BUD is available in many formulations in the market, such as ileal release formulation, tablets, and enema, etc. In the account of the beneficial effects of nano-sponge, and due to drawbacks of traditional topical drug delivery, and absence of availability of nano-sponges based DDS; present research work was intended to figure out and assess novel nano-sponges based BUD formulation for its promising effects in the treatment of IBD.^18^

## Materials and Methods

### Experimental design

BUD was obtained from Astra Zeneca; Dibutyl phthalate and PVA were obtained from Central Drug House Pvt. Ltd Mumbai, India. Eudragit S-100 was provided by Evonik Pharma, Mumbai, India., and Hualien’s Fine Chemicals, Mumbai, India, respectively. Demineralized and fresh distilled water was prepared as required. Analytical grade materials and reagents were used for this study. The required materials were procured from the diver sources.

### Characterization of BUD 

The pure drug BUD was subjected to characterization for the purity and the important characterizations were mentioned below.

### Determination of λ max of BUD 

Ten milligrams of BUD were weighed and added in 10 ml of 7.4 pH buffer solution and 0.1 N HCl independently in a volumetric flask of 10 ml. The obtained solution 1000 µg/mL and from the above solution, 1 mL solution was carrying forwarded in 10 mL volumetric flask and volume was made with (7.4 pH) buffer solution and 0.1 N HCl separately. Required dilution was prepared to get a concentration range of 5-25 μg/mL for both solvents. The solution was observed in the 200-400 nm range in the UV light (Labindia-3000+). The concentration Vs. absorbance graph was plotted and the calibration curve was made.^19^

### FTIR spectra of BUD 

An IR spectrum of the drug was measured by KBr method using Fourier transform infrared (FTIR). The correction of the baseline was made by using dried potassium bromide pellet. The potassium bromide-pellet was set up by crushing 3-5 mg of the physical blend of medication and KBr in the hydraulic pressure. The obtained pellet was kept in the IR compartment and measured at wavelengths 4000 cm^-1^ to 400 cm.^20^

### Differential scanning calorimetry of BUD

The thermogram of the drug was recorded using a DSC. The drug sample was weighed in the hermetically closed aluminum pans. The sample was heated over a temperature of 50-400°C in an atmosphere of nitrogen (200 mL/min) at a constant rate of 10°C/min, with alumina being the reference standard.^21^

### Preparation of nano-sponges

#### Formulation design

Each numeric factor is set to 3 levels. If categoric factors are incorporated, the Box-Behnken design will be copied for each combination of the categoric factor levels. These designs have fewer runs than 3-level factorials design. The formulation was designed by using DOE 11.0. The design was run for 29 total runs.

### Method of preparation

The nano-sponges containing BUD were formulated by a method called the quasi-emulsion solvent diffusion. The accurately weighed amount of polymethyl-methacrylate, Eudragit S-100 in different ratios with dibutyl phthalate (1% w/v) were dissolved in 10 mL of dichloromethane: methanol (50:50). Dibutyl phthalate was incorporated to increase the polymer plasticity. BUD was dissolved in this mixture. At the next, 0.5-1.5% w/v solution with distilled water was prepared as dispersing media. The previously prepared solution of polymers and drug was added gradually in PVA solution and stirring was kept constant for 2 hours. After complete evaporation of solvent from polymer droplets, nano-sponges were formed, which were centrifuged at 4000 rpm for collection and followed by 3 times washing. The solvent was slowly removed to form the nano-sponges. The aqueous suspension of nano-sponges was lyophilized and stored in a tightly sealed container until further analysis. The optimization of the formulation of BUD loaded nano-sponges was given in Table 1.^22^

**Table 1:** Formulation design for budesonide nano-sponges

**Std**	**Run**	**Factor 1**	**Factor 2**	**Factor 3**	**Factor 4**	**Response 1**	**Response 2**	**Response 3**
		**A: Eudragit S-100: PMMA**	**B: PVA**	**C: Budesonide**	**D: Stirring Speed**	**Z-Average**	**% EE**	**PDI**
		**% w/v**	**% w/v**	**% w/w**	**rpm**	**nm**	**%**	
1	1	0.2	0.5	20	2000	578	37	0.87
15	2	0.4	0.5	30	2000	593	53	0.92
9	3	0.2	1	20	1000	515	42	0.73
26	4	0.4	1	20	2000	560	51	0.85
24	5	0.4	1.5	20	3000	480	48	0.64
25	6	0.4	1	20	2000	562	50.6	0.84
10	7	0.6	1	20	1000	590	55.9	0.93
3	8	0.2	1.5	20	2000	510	35	0.62
22	9	0.4	1.5	20	1000	475	52	0.65
4	10	0.6	1.5	20	2000	580	53.5	0.87
19	11	0.2	1	30	2000	538	32	0.68
29	12	0.4	1	20	2000	558	36	0.87
28	13	0.4	1	20	2000	556	37	0.88
17	14	0.2	1	10	2000	520	28	0.78
18	15	0.6	1	10	2000	585	45.8	0.72
27	16	0.4	1	20	2000	557	51	0.85
11	17	0.2	1	20	3000	516	26	0.57
23	18	0.4	0.5	20	3000	515	47.4	0.88
6	19	0.4	1	30	1000	640	50.9	0.91
5	20	0.4	1	10	1000	634	34	0.89
8	21	0.4	1	30	3000	504	49	0.84
12	22	0.6	1	20	3000	525	52.3	0.74
7	23	0.4	1	10	3000	498	46	0.82
2	24	0.6	0.5	20	2000	538	48.7	0.83
16	25	0.4	1.5	30	2000	493	52	0.65
14	26	0.4	1.5	10	2000	491	43	0.59
21	27	0.4	0.5	20	1000	585	46	0.74
13	28	0.4	0.5	10	2000	575	42	0.72
20	29	0.6	1	30	2000	565	52.7	0.86

%EE: percentage entrapment efficiency; PDI: polydispersity index.

### Evaluation of nano-sponges

#### Determination of PDI and particle size using zeta-sizer

The polydispersity index (PDI) and average particle size of prepared nano-sponges were examined by using zeta sizer (DTS were.4.10, Horriba instrument, India). Dilution of nano-sponges preparation was done with deionized water (1:9 v/v) and analyzed for the average size and PDI.^23^

### Vesicle morphology by using scanning electron microscopy

Vesicle morphology was determined by using scanning electron microscopy (IISER, Bhopal). The nano-sponges were fixed on supports with carbon-glue and coated with gold using a gold sputter module in a high-vacuum evaporator. Samples were visualized by SEM at 10 kV.^24^

### Determination of drug content using UV spectrophotometry

The quantity of drug incorporated in the nano-sponges was investigated by using a UV spectrophotometer. The nano-sponges were incubated with PBS (pH 7.4), for 48 hours. After incubation, it was centrifuged at 10,000 rpm for 30 minutes and the supernatant was diluted 10 times before analysis into the UV spectrophotometer system and it was read at 252 nm.^25^

### In vitro drug release from nano-sponges

*In vitro*, drug release studies were investigated in the presence of rat’s caecal contents. Albino Wistar rats (200-250 g) were taken and kept on normal diet conditions. These rats were also given 1 mL of 2% w/v mixture of polymethylmethacrylate and eudragit for 7 days for an enzyme. The rats were sacrificed, and a ligature was made before and after the caecum by opening the abdomen and subsequently the caecum was removed and transferred in PBS (pH 7.0), which was previously bubbled with CO_2_. The caecum bag was opened, the contents were weighed and homogenized to PBS (pH 7.0) to prepare 1%, 2%, and 4% of the caecal solution and then it was utilized as simulated colonic fluid. The suspension was filtered through cotton wool and sonicated for 20 minutes at 4^o^C to disrupt the bacterial cells, and then centrifuged at 2000 rpm for 20 minutes. The drug release study of nano-sponges was carried out in sealed glass vials at 37±0.1^o^C. nano-sponges (100 mg) were weighed and filled in gelatin capsules and taken into a beaker containing 100 ml of PBS (pH 7.0) containing 1%, 2%, and 3% rat’s caecal contents. Simultaneously, a similar experiment was performed containing simulated colonic fluid without enzyme induction. The filtrate was examined using a UV spectrophotometer.^26,27^

### In vivo anti-ulcer activity in animal 

#### Experimental animals 

Male Wistar rats were procured from the laboratory animal facility. They were housed in standard temperature and relative humidity. All animals were provided with a standard pellet diet and water *adlibitum*.

### Induction of experimental colitis

The rats were divided into various experimental groups, and they were given mild ether anesthesia. A rubber catheter was embedded rectally into the colon such that the tip was 8 cm proximal to the anus, roughly at the splenic flexor. 2, 4, 6-trinitrobenzenesulfonic acid (TNBZ) (30 mg) added in 50% ethanol (vol/vol) was ingrained within the lumen of the colon through the rubber catheter (complete volume, 0.25 mL). The dose of TNBZ (30 mg) was used in consequent experiments. In control rats, it was given 0.25 mL of either 50% ethanol alone or 30 mg of TNBZ in 0.9% saline, or 0.9% saline alone.

The standard group received the 9 µg/kg of the marketed formulation of BUD for 7 days along with 30 mg TNBZ and the optimized formulation of BUD was given to the test group along with 30 mg TNBZ.^28-30^

The animals were randomly divided into four groups (n=6)

Group I: Control rats: received vehicle (1 mL/kg).

Group II: Colitis control (received 30 mg TNBZ)

Group III: Animals with induced BUD and treated with the standard marketed drug (9 mg/kg).

Group IV: Animals with induced BUD and treated with the optimized formulation of BUD nano-sponges. The animals received treatment orally for a continuous 7 days (once daily). Animals were sacrificed with standard system 24 hours after the last treatment. A section of colon 8 cm long was removed and assessed.

### Determination of colon/body weight ratio (C/B ratio)

The colon/body weight ratio was determined as an index of colonic tissue edema. The rats were euthanized, and the stomach area was opened, and the opening distal colon was done longitudinally with the mesenteric edge. The distal colon samples were washed with isotonic saline. An 8 cm segment showing gross pathological changes was weighed for determination of ratio.^31,32^

### Disease activity index (DAI)

The clinical activity of the disease was assessed by utilizing a qualitative disease activity index (DAI) scoring technique consistently by consolidating the scores of bodyweight loss, stool consistency and fecal bleeding.^33,34^

### Change in body weight

The disease activity index was determined as the sum of the bodyweight loss which was scored as Table 2.

**Table 2:** Bodyweight loss scoring, faucal bleeding scoring, stool consistency scoring^35^

**Score**	**Bodyweight** **scoring**	**Faucal bleeding** **scoring**	**Stool consistency** **scoring**	**Microscopic damage** **score**
0	None	Negative hemoccult	Well-formed pellets	No damage
1	1-5%	--	--	Localized hyperemia with no ulceration
2	5-10%	Positive hemoccult	Loose stools	Linear ulceration with no significant inflammation
3	10-20%	--	--	Linear ulcers with inflammation at one site
4	Over 20%	Gross bleeding	Diarrhea	Two or more sites of ulceration and/or inflammation
5	--	--	--	Two or more sites of ulceration and inflammation of one major site of inflammation and ulceration >1 cm along with length

### Fecal bleeding

The fecal bleeding was calculated based on the presence or absence of blood in feces.

### Measurement of myeloperoxidase (MPO) activity

The distal colon sample (200 mg) was cut and added 1 mL of hexadecyl trimethyl ammonium bromide (HTAB) buffer (0.5% HTAB in 50 mM phosphate buffer, pH 6.0) on ice, moved to a test tube and homogenized (multiple times for 30 s each on ice). Homogenate was centrifuged for 15 minutes at 10 000 rpm. The supernatant was assayed by the UV chamber for MPO action. 0.1 ml of supernatant was mixed with 2.9 mL of 50 mM phosphate buffer (pH 6.0) having O-dianisidine hydrochloride (0.167 mg/mL) and hydrogen peroxide (0.0005%). The change in absorbance at 460 nm was estimated.^36,37^

### Histopathological evaluation 

A tiny specimen of the GI wall from each animal was fixed in a 10% buffered formalin solution followed by tissue dehydration with alcohol and xylene. At that point, all samples were fixed in paraffin wax and segmented into 5-μm slides before staining. The slides were stained with hematoxylin and eosin (H&E).^38^

### Statistical analysis

The data was shown in mean ± SD. Analyzed by one-way analysis of variance (ANOVA) followed by Dennett’s test. The data were analyzed with GraphPad Prism software. The criterion for statistical significance was *P* < 0.01 or *P* < 0.05.

## Results and Discussion

### Determination of λmax of BUD

BUD showed a linear relationship with the correlation coefficient of 0.998 and 0.999 in the concentration range of 5-25 μg/mL in phosphate buffer pH 7.4 and 0.1 N HCl respectively. The absorption maxima of drug BUD were found to be 252 nm, which shows the purity of the drug.

### FTIR spectra of BUD 

**Figure 1:**
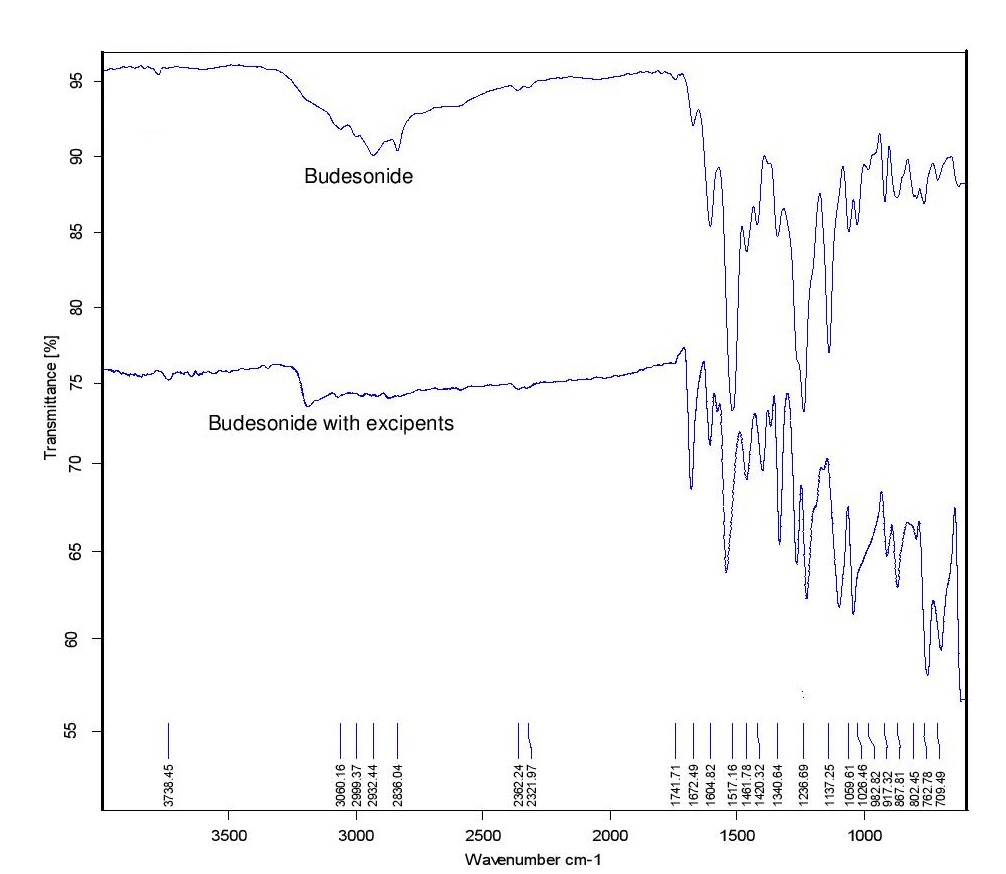
FTIR of Budesonide and Budesonide with excipients.

BUD presented a characteristic peak at 3738.45 cm^-1^ due to OH stretching, 2836 cm^-1^ was due to CH stretching vibration. In at 1672.49 cm^-1^ presenting C=O stretching vibration, peak at 1413.05 cm^-1^ was due to C-H bending (aromatic). No new peak observed confirming the authenticity of the sample shown in Figure 1.

### Differential scanning calorimetry of BUD

**Figure 2:**
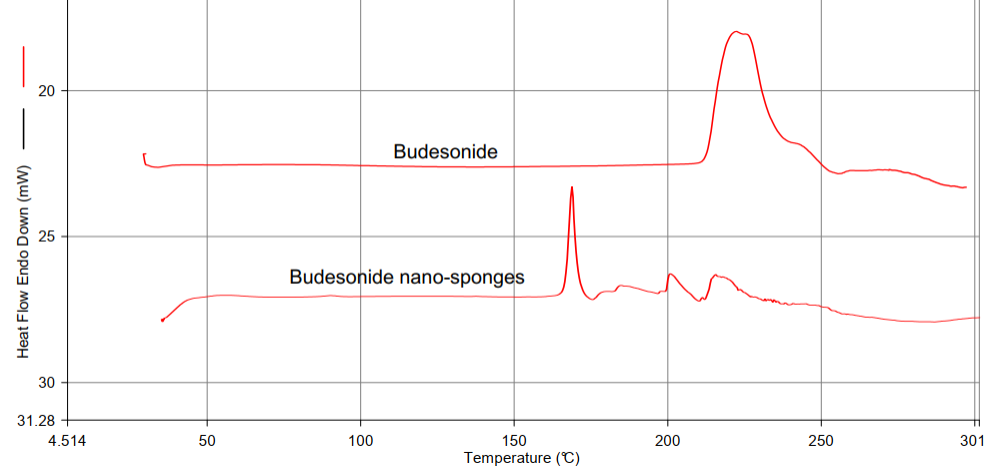
Differential scanning calorimetry of budesonide drug excipient compatibility study by DSC.

A sharp endothermic peak was observed at 225°C concerning the melting point of the drug shown in Figure 2.

### Drug excipient compatibility study by DSC

The compatibility was defined for the interaction of BUD and the excipients. It was determined using differential scanning calorimetry (DSC). The DSC thermogram of BUD exhibited a melting point at 225°C. The mixture of drug and cholesterol, which was kept in an accelerated condition of 40°C/75% relative humidity for 30 days and subjected to DSC analysis. The characteristic melting point of BUD not deviated from 225°C that predicts there no interaction between drug and excipients shown in Figure 2.

### DOE study and evaluation

#### Evaluation of nano-sponges

#### Determination of Particle Size, PDI using zeta-sizer

The particle size and polydispersity were evaluated using zeta sizer.

### Scanning electron microscopy

**Figure 3:**
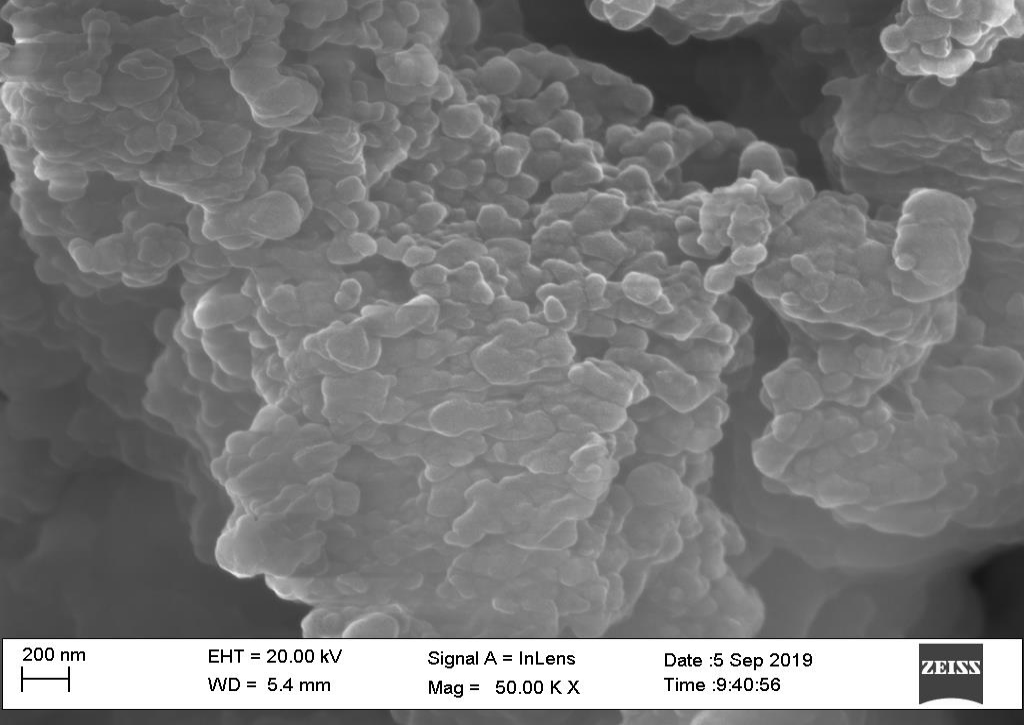
Scanning electron microscopic images of formulated nano-sponges.

Nano-sponges were determined by using scanning electron microscopy analysis to check their surface topography and morphology. The captured scanning electron microscopy pictures of nano-sponges (Figure 3) SEM photomicrographs reflected that nano-sponges formed were porous. Pores were induced by the diffusion of solvent from the surface of nano-sponges. Furthermore, it was shown that the distinctive internal structure comprised of a spherical cavity.

### Determination of drug content 

The quantity of drug present in the nano-sponges was determined by the % drug content study in the formulation. The drug content is both batches were found to be 96.89% and 94.73%.

### In vitro release of BUD from nano-sponges

**Figure 4:**
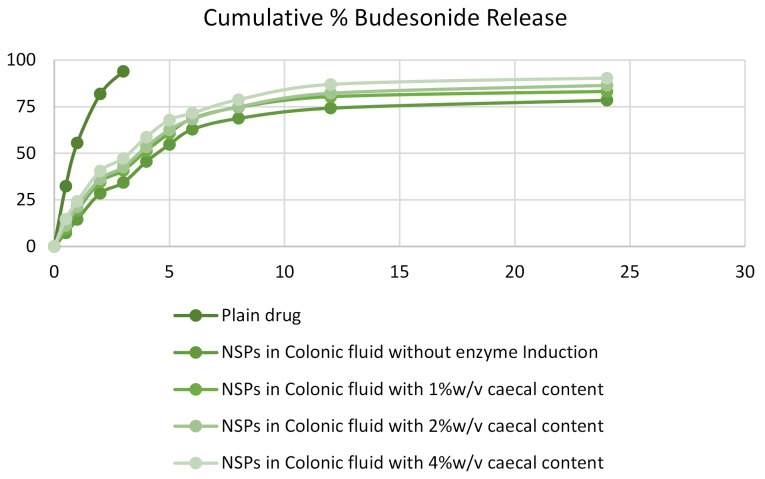
Cumulative % budesonide release from different optimized batches.

The *in vitro* release from an optimized formulation of nano-sponges was determined to calculate the *in vitro* drug release from prepared formulation. The results obtained are as shown in Figure 4.

### In vivo anti-ulcer activity in animals

#### The C/B mass ratio

Rats were observed for symptoms of UC, the majority of rats from groups III and IV did not show a thick bowel with ulcerations, thrombosis with endothelial proliferation. The injured areas were much reduced; in some animals, it was similar to the normal group. The C/B mass proportion after intracolonic administration of TNBZ was altogether higher contrasted with normal (P < 00.001). After the oral administration of optimized formulation, the C/B proportion was less contrasted with the colitis control group. The reduction in C/B proportion is because of the anti-inflammatory action of the optimized formulation. A list of colonic tissue edema (colon/bodyweight proportion) is comparable to that of the standard. The index of colonic tissue edema (colon/body weight ratio).

### Disease activity index (DAI)

**Figure 5:**
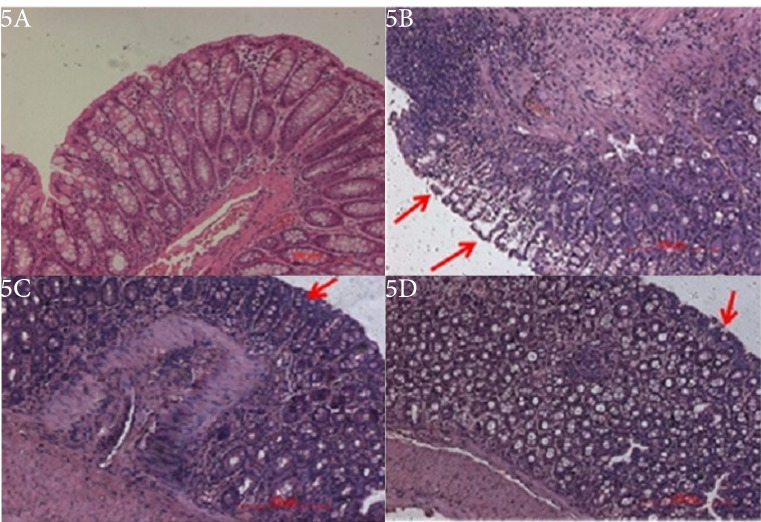
Effect of Budesonide marketed and optimized formulation on colonic histopathology.

A dose-dependent effect of BUD marketed formulation and the optimized formulation were evaluated as compared to the TNBZ group. The assessment of the clinical activity scores like stool consistency, stool blood, and weight loss was checked for disease severity on day 7. Clinical activity score was seen in the colitis control group, which shows severe disease progression of acute colitis with a DAI score of 3.9. Alternatively, the group treated by marketed formulation displayed reduced DAI scores 2.6, respectively. The optimized formulation-treated group showed reduced DAI scores 3.0, Furthermore, the control group observed decreased in the body weight, rectal bleeding while, treated groups retained weight loss and stool consistency, Also, colon tissues from experimental groups were collected and measured for their colon length. The optimized formulation had a therapeutic effect and showed a decrease in the colon length, which is associated with colonic inflammation (Figure 5).

### Assessment of macroscopic damage score

Animals treated with BUD optimized formulation and the standard drug displayed a significant enhancement of the wasting disease compared with animals treated with TNBZ alone, as analyzed by macroscopic activity score and body weight change of rats. The colons of TNBZ rats showed marked edema, hyperemia, and inflammation, while the colons of normal control rats treated with saline alone indicated no or a slight inflammation (Figure 5). Treatment along with BUD optimized formulation and standard treatment reduced both hyperemia and inflammation in the colons.

### Measurement of MPO activity

**Figure 6:**
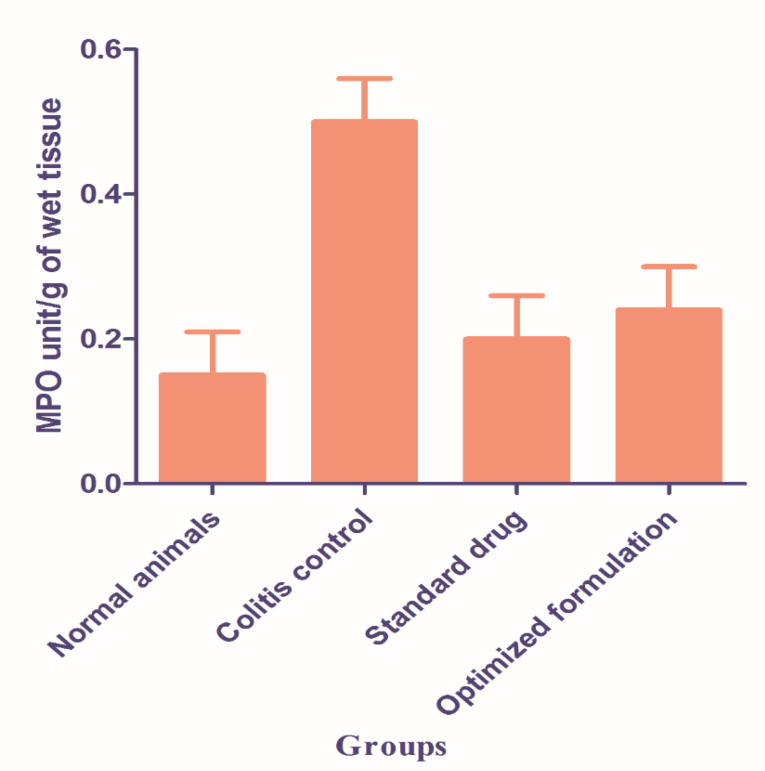
Effect of budesonide marketed and optimized formulation on myeloperoxidase activity in colon tissue.

The activity of the MPO enzyme has been essentially decreased after the administration of both standard and optimized formulations of BUD. An MPO activity indicates a positive correlation with histopathologic observations. The presence of increased neutrophils infiltration into the colon tissue in TNBZ- alone treated group versus decreased neutrophil infiltration in the BUD treated group. The presence or absence of neutrophil infiltration correlates with the MPO activity of colon tissue given in Figure 6. Statistical significance was determined by one-way ANOVA followed by Dunnet’s Comparison Test Values are statistically significant, (P < 00.001) and shows improvement in the standard drug and optimized formulation

### Histopathological evaluation

The severity of colon tissue damage was assessed by histological analysis by using Hematoxylin and Eosin staining. The histological assessment of the colons from normal control (Figure 5A) showed normal mucosal epithelium cells along with submucosal glands observed with no ulceration or inflammation. The control rats showed intact epithelium and mucosa with retained cellular integrity and no infiltration of leukocytes. Tissue sections from the rats injected with TNBZ alone (Figure 5B) revealed demolition of crypt structure with loss of the goblet cells, disruption of the epithelium layer, moderate to severe submucosal inflammation along with the infiltration of the inflammatory cells, massive infiltration of the inflammatory cells with the cryptic abscess into the colon tissue and also showed severe lesions, with complete loss of colonic epithelial cells and presence of inflammatory cell infiltration. The colon tissue sections from standard formulation and TNBZ- induced rats (Figure 5C) showed lower evidence of cryptic harm with the saved goblet cells and the epithelial lining, mild sub mucosa hemorrhages together with the mild foci of infiltration of the inflammatory cells in the submucosal region in the colon tissue. However, optimized formulations treatment in TNBZ- induced mice (Figure 5D) revealed no damage to the colon with decreased signs of inflammation into colonic tissue, preserved epithelial layer and mucosal epithelial cells, crypt structure with goblet cells are normal or no inflammation.

## Conclusion

Nano-sponges based BUD system was developed successfully by using a quasi-emulsion solvent diffusion method for prolonged transport of drugs for an extended period to decrease application frequency allied to the standard marketed formulation and to enhance bioavailability and safety. The analytical characterization showed good purity of the drug. *In vitro* drug release showed a good release profile of prepared optimized-sponges formulation.

The *in vivo* test revealed that optimized formulation prevents the morphological and functional alteration of the colonic tissues by improving the redox balance in the colon. It has shown an effective decrease in the colon to MPO activity, Clinical activity score, and body weight ratio suggesting its protective activity. The histopathology also suggested the efficacy of optimized formulation in the protection of IBD.

Thus, the nano-sponges based on the delivery system developed and assessed in the current research approach was seemed to be auspicious concerning preventing BUD, the disease and other colonic diseases along with practical utilization in the pharmaceutical field.

## Ethics Issues

The animals were maintained in conformity with the regulations laid down by the Committee for the Purpose of Control and Supervision of the Experiments on Animals (CPCSEA) constituted under the Prevention of the cruelty to animals act, 1960, Ministry of Environment and Forests, Government of India. Experimental protocol was approved by IAEC (Protocol approval No. IAEC/CPCSEA/IPE/2018-9).

## Conflict of Interest

The author has no conflict of interest in this research.

## Acknowledgments

The author Amarjit Salunke is grateful to his research guide Dr. Neeraj Upmanyu for helping him to carry out this research.
